# Long Noncoding RNA HOTAIR Is a Prognostic Marker for Esophageal Squamous Cell Carcinoma Progression and Survival

**DOI:** 10.1371/journal.pone.0063516

**Published:** 2013-05-23

**Authors:** Xiao-Bin Lv, Gui-Yong Lian, Hao-Ran Wang, Erwei Song, Herui Yao, Ming-Hui Wang

**Affiliations:** 1 Department of Thoracic-Cardiac Surgery, Sun Yat-sen Memorial Hospital, Sun Yat-sen University, Guangzhou 510120, China; 2 Breast Tumor Center, Department of Breast Surgery, Sun Yat-sen Memorial Hospital, Sun Yat-sen University, Guangzhou 510120, China; 3 Medical Research Center Sun Yat-Sen Memorial Hospital, Sun Yat-Sen University, Guangzhou 510120, China; Health Canada, Canada

## Abstract

**Background:**

It is currently unclear whether the expression of HOX transcript antisense RNA (HOTAIR) correlates with the progression of esophageal cancer. The aim of this study was to examine HOTAIR expression in patients with esophageal squamous cell cancer (ESCC) and explore its clinical significance.

**Methods:**

Differences in the expression of HOTAIR were examined via in situ hybridization (ISH) and quantitative reverse transcriptase PCR (qRT-PCR). The prognostic significance was evaluated using Kaplan–Meier and Cox regression analyses. Proliferation, colony formation and migration assays were performed in ESCC cell lines to determine the function of HOTAIR in the progression of ESCC in vitro.

**Results:**

A notably higher level of HOTAIR expression was found in ESCC tissues. High expression levels of HOTAIR in ESCC patients correlated positively with clinical stage, TNM classification, histological differentiation and vital status. HOTAIR expression was found to be an independent prognostic factor in ESCC patients. ESCC patients who expressed high levels of HOTAIR had substantially lower overall 5-year survival rates than HOTAIR-negative patients. In vitro assays of ESCC cell lines demonstrated that HOTAIR mediated the proliferation, colony formation and migratory capacity of ESCC cells.

**Conclusion:**

HOTAIR is a potential biomarker for ESCC prognosis, and the dysregulation of HOTAIR may play an important role in ESCC progression.

## Introduction

Esophageal cancer (EC) ranks as the ninth most common malignancy and the sixth most frequent cause of cancer deaths worldwide, with occurrence rates that vary greatly by geographic location [1]. Approximately half of the EC cases that are newly diagnosed each year occur in China [2]. Histologically, esophageal squamous cell carcinoma (ESCC) and esophageal adenocarcinoma (EAC) comprise more than 90% of ECs [3]. The majority of ESCC patients present with advanced metastatic disease at the initial diagnosis, and the overall 5-year survival rate is <10%.

Although findings from molecular biology studies have improved our general understanding of the pathogenesis of ESCC, the appropriate biomarkers for high-risk population screening, clinical diagnosis and prognosis have not yet been identified. Therefore, it is imperative to seek more effective biomarkers for the early diagnosis of ESCC. Previous reports have shown that genetic changes frequently associated with the development of esophageal cancer include the p53 mutation, p16 inactivation, cyclin D1 amplification and c-Myc, GOLPH3 or EGFR overexpression [4]–[6].

Recently, a number of studies have shown that the expression of many long noncoding RNAs (lncRNAs) are dysregulated in various cancers and that these lncRNAs play important roles in tumorigenesis and tumor progression [7]–[9]. One example of such an oncogenic lncRNA is the HOX transcript antisense RNA (HOTAIR), which is expressed from the *HOXC* locus. HOTAIR was initially discovered as a repressor of the *HOXD* genes [10], [11]. Subsequent studies suggested HOTAIR induced genome-wide retargeting of PRC2, leading to H3K27me3, and promoted metastasis of breast cancer by silencing multiple metastasis suppressor genes [12]. Using a series of HOTAIR deletion mutants, Tsai and colleagues identified that HOTAIR serves as a scaffold tether two distinct complexes PRC2 and LSD1 and coordinates targeting of PRC2 and LSD1 to chromatin for coupled histone H3 lysine 27 methylation and lysine 4 demethylation [11]. Phosphorylation of the PRC2 complex element EZH2 up-regulates its binding to HOTAIR [13]. Recently, researchers have found that HOTAIR is significantly overexpressed in a variety of tumors and is able to induce the proliferation and metastasis of these tumors [14]. Clinically, overexpression of HOTAIR is a powerful predictor of overall survival and progression for several cancers including pancreatic cancer [15], hepatocellular carcinoma [16], [17], colon cancer [18], nasopharyngeal carcinoma laryngeal [19], squamous cell carcinoma [20], breast cancer [21] and gastrointestinal stromal tumors [22].

However, the relationship between HOTAIR and esophageal cancer remains unclear. The objective of this study was to investigate the expression of HOTAIR in ESCC and to further explore the clinical significance of HOTAIR expression. In this study, quantitative reverse transcriptase PCR (qRT-PCR) and in situ hybridization (ISH) were used to examine the HOTAIR expression. Additionally, the correlation of HOTAIR expression with ESCC-specific clinicopathological features was assessed.

## Materials and Methods

### Patient and Tissue Specimens

The patients accepted into this study were diagnosed with ESCC between 1996 and 2005 at the Sun Yat-Sen Memorial Hospital and underwent esophageal cancer resection surgeries prior to the administration of chemotherapy (Table 1). ESCC and adjacent tissues were obtained from the resected tumors and adjacent noncancerous esophageal tissues, respectively, and were confirmed by a pathological assessment. Written informed consent was obtained from all patients, and the protocol was approved by the Research Ethics Board at Sun Yat-Sen Memorial Hospital. ESCC specimens were staged in accordance with the American Joint Cancer Committee/Union Internationale Contre le Cancer (UICC/AJCC) classification guidelines.

**Table 1:** Correlation between the clinicopathologic features and expression of HOTAIR.

Characteristics	HOTAIR in situ hybridization (%)		P
	No. of low expression	No. of high expression	
Age(y)			
**≤60**	26 (44.1)	33 (55.9)	0.272
**>60**	19 (52.9)	15 (47.1)	
Gender			
Male	24 (42.9)	32 (57.1)	0.189
Female	21 (56.8)	16 (43.2)	
Clinical stage			
I	6 (100.0)	0 (0.0)	0.001
II–III	37 (50.7)	36 (49.3)	
IV	2 (14.3)	12 (85.7)	
T classification			
T1	9 (100.0)	0 (0.0)	0.000
T2–T3	34 (48.6)	36 (51.4)	
T4	2 (14.3)	12 (85.7)	
N classification			
N0	28 (59.6)	19 (40.4)	0.005
N1	17 (37.0)	29 (63.0)	
Metastasis			
M0	41 (48.7)	38 (51.3)	0.029
M1	4 (28.9)	10 (71.1)	
Differentiation			
Well	29 (61.7)	18 (38.3)	0.022
Moderate	14 (38.9)	22 (61.1)	
Poor	2 (20.0)	8 (80.0)	

### Real Time Quantitative PCR

Total RNA from cancerous and noncancerous specimens or cell lines was extracted with the Trizol reagent (Applied Biosystems Inc, USA) according to the manufacturer’s instructions. cDNA was obtained by reverse transcribing the total RNA with a TaqMan Reverse Transcription Kit (Applied Biosystems Inc, USA). The following primer sequences were used to amplify HOTAIR: forward, GGTAGAAAAAGCAACCACGAAGC and reverse, ACATAAACCTCTGTCTGTGAGTGCC. Amplification and analysis were performed on the Roche LightCycler480 Real-Time PCR System. Beta-actin was used as an internal control, and HOTAIR values were normalized to those of beta-actin. The following primer sequences were used to amplify beta-actin: forward, TGAAGTGTGACGTGGACATC and reverse, GGAGGAGCAATGATCTTGAT.

### Oligonucleotide Transfection

Small interfering RNAs (siRNAs) to specifically target human HOTAIR were designed according to previously validated oligonucleotides [12] and were synthesized by GenePharma (Shanghai, China). SiRNA against GFP (si-GFP) bought from GenePharma (Shanghai, China) were used as the negative control. The siRNAs were transfected into cells at a working concentration of 50 nmol/L with the RNAiMAX reagent (Applied Biosystems Inc, USA) according to the manufacturer’s instructions.

### MTT and Colony Formation Assays

For cell proliferation assays, the cells that had been transfected with siRNAs for 24 h were reseeded into 96-well plates at 1.5×10^3^ cells/well in a final volume of 150 µL and were incubated for the indicated time periods. The effect of HOTAIR siRNA on cell growth and proliferation was determined with an MTT assay as described previously [23].

For colony-forming assays, the cells were reseeded at 48 h post-transfection into 6-well plates at 500 or 1,000 cells per well. The culture medium was replaced every 3 days. After a 2-week incubation at 37°C, the cells were washed twice with PBS, fixed and stained with 0.5% crystal violet. The numbers of colonies were counted by microscopy.

### Transwell Assay

Transwell assays were performed in modified Boyden chambers with 8-µm pore filter inserts in 24-well plates (BD Transduction). Briefly, 10^5^ cells in serum-free Dulbecco’s Modified Eagle Medium (DMEM) were added to the upper chambers (BD Biosciences) of the inserts of a 24-well culture plate. Fetal bovine serum (FBS), a chemoattractant, was added to the lower chambers. After 8 h, the non-filtered cells were gently removed with cotton swabs. The cells that had passed through the filters to the lower sides of the chambers were stained with crystal violet, air-dried and photographed.

### In situ Hybridization and Data Analyses

HOTAIR expression was examined by in situ hybridization in ESCC and non-ESCC paraffin-embedded sections. Briefly, after dewaxing and rehydration, the samples were digested with proteinase K, fixed in 4% paraformaldehyde and hybridized with a 5′ digoxin-labeled LNA™-modified HOTAIR probe (Exiqon) overnight at 55°C. The samples were then incubated overnight at 4°C with an anti-digoxin monoclonal antibody (Roche Applied Science). The sections were stained with nitro blue tetrazolium/5-bromo-4-chloro-3-indolylphosphate (NBT/BCIP) in the dark, mounted and observed. HOTAIR-positive staining (in blue) was primarily detected in the cytoplasm of cells. The staining scores were determined by microscopy on the basis of both the intensity and proportion of HOTAIR-positive cells in 10 random fields under a 40× objective. The proportion of positively stained tumor cells was graded according to the following: 0, no positive cells; 1, <10% positive cells; 2, 10%–50% positive cells; and 3, >50% positive cells. The staining intensity of the cells was graded according to the following: 0 (no staining), 1 (light blue), 2 (blue) and 3 (dark blue). The staining index (SI) was calculated as SI = staining intensity×proportion of positively stained cells. The expression of HOTAIR was evaluated using the SI and was scored as 0, 1, 2, 3, 4, 6 or 9. An SI score of 6 was used as a cut-off value based on a measurement of heterogeneity with the log-rank test statistic with respect to overall survival, and the expression levels of HOTAIR were defined as high (SI ≥6) or low (SI <6). In addition, the in situ hybridization signals for HOTAIR expression in tumors and normal tissues were quantified by mean optical density (MOD) using the AxioVision Rel.4.6 computerized image analysis system and the automatic measurement program (Carl Zeiss, Oberkochen, Germany) as previously reported [24]. Briefly, the stained slides were evaluated at 200× magnification with the SAMBA 4000 computerized image analysis system and the Immuno 4.0 quantitative program (Image Products International, Chantilly, VA), and 10 randomly selected fields for each specimen were analyzed to determine the MOD, which represented the strength of the staining signal per positive pixel. The areas and numbers of positively stained tumor cells were also considered in this program to eliminate the impact of cell density on the MOD value. A negative control was used with each staining set to serve as background subtraction in the quantitative analysis. The representative staining fields for each specimen were analyzed and scored independently by two observers who were blinded to each other and to the diagnoses of the specimens.

### Statistical Analyses

All statistical analyses were performed with the SPSS 16.0 statistical software package (SPSS Inc., Chicago, IL). A chi-squared test was used to analyze the relationship between HOTAIR expression levels and the clinicopathological characteristics. Survival curves were plotted according to the Kaplan-Meier method and were compared using the log-rank test. The survival data were evaluated using univariate and multivariate Cox regression analyses. In all cases, *P*<0.05 was considered significant.

## Results

### Increased Expression of HOTAIR in ESCC Cells

**Figure 1:**
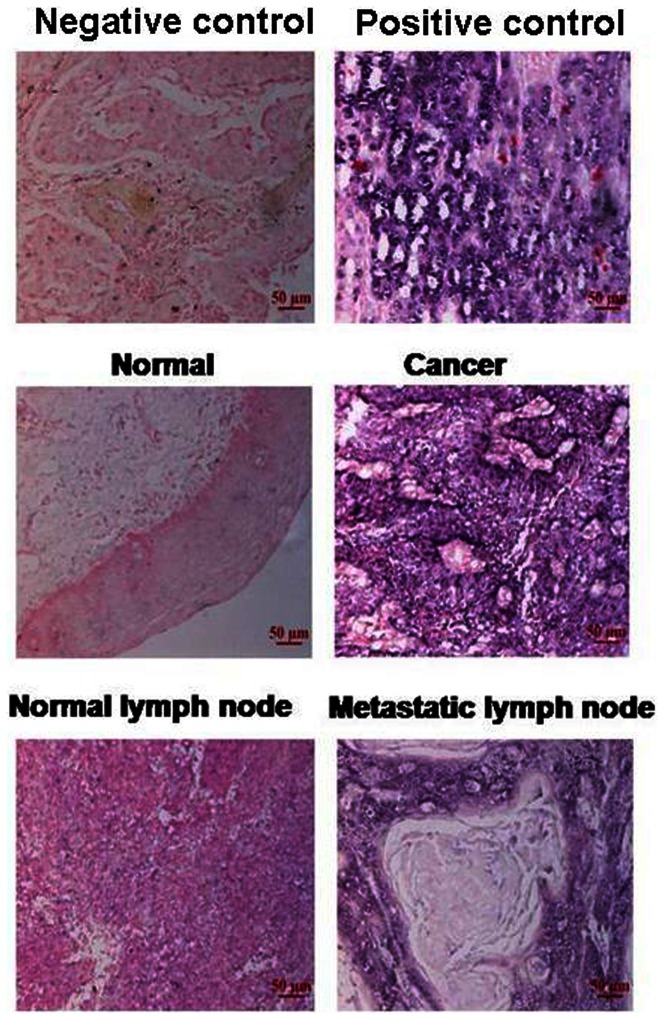
Increased HOTAIR expression was detected in ESCC tissues and metastatic lymph nodes. Representative images of in situ hybridization for HOTAIR in non-ESCC tissue, ESCC tissue, a normal lymph node and a metastatic lymph node.

To examine whether the expression of HOTAIR is linked to the clinical progression of ESCC, a total of 93 paired paraffin-embedded noncancerous and ESCC tissues were subjected to ISH staining with a digoxin-labeled probe against HOTAIR. Scattered, HOTAIR-specific staining was observed in the cytoplasm of carcinoma cells in 48 of the 93 cases, whereas no staining was observed in the normal ESCC cells (Figure 1).

**Figure 2:**
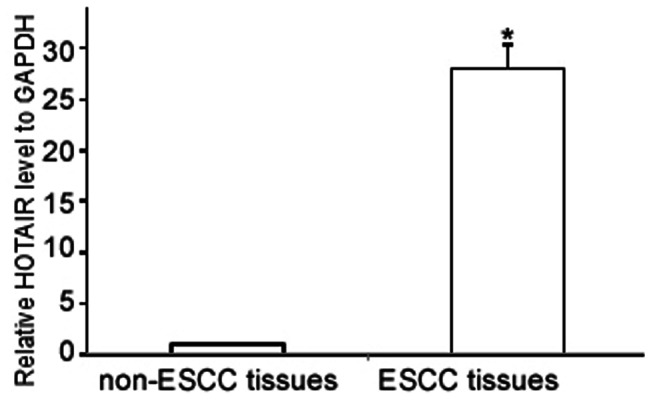
HOTAIR expression levels were upregulated in ESCC tissues compared with non-ESCC tissues as detected by qRT-PCR. Total RNA from 30 paired ESCC and non-ESCC specimens was extracted with the Trizol reagent, and HOTAIR expression levels were evaluated by qRT-PCR. The results are expressed as the means ± SD; * *P*<0.001 compared with non-ESCC tissues.

Next, we correlated the HOTAIR expression levels with the clinicopathological statuses of patients with ESCC (Table 1). The expression levels of HOTAIR were upregulated in the tumors of patients with higher tumor burdens; these patients were characterized as having a larger tumor size (*P* = 0.000, Table 1), more advanced clinical staging (*P = *0.001, Table 1) and increased lymph node tumor burden (*P* = 0.029, Table 1). Furthermore, HOTAIR expression in poorly differentiated ESCC tissues was significantly higher than that in well or moderately differentiated ESCC tissues (*P* = 0.022, Table 1). Statistical analyses showed no relationship between patient gender or age and the expression level of HOTAIR. To further evaluate whether HOTAIR upregulation was linked to NPC clinical progression, HOTAIR levels in 30 pairs of freshly frozen ESCC and adjacent noncancerous tissues were evaluated using qRT-PCR. The mean expression level of HOTAIR in ESCC samples was 28-fold higher than the mean level in the adjacent noncancerous tissue samples (Figure 2). Consistent with the observations from the ISH, the enhanced expression levels of HOTAIR correlated with a higher tumor burden, more advanced clinical staging, increased lymph node tumor burden and poor differentiation (Table 2). Taken together, these observations indicate that the progression of ESCC is associated with increased HOTAIR expression.

**Table 2:** Correlation between the clinicopathologic features and expression of HOTAIR.

Characteristics	Relative HOTAIR level compared with non-cancerous samples evaluated by qRT-PCR		
age(y)	Number	2^−△△Ct^	
≤60	16	28.785	0.901
>60	14	29.211	
gender			
Male	16	30.107	1
Female	14	26.834	
Clinical stage			
I	4	9.675	0.001
II–III	24	30.557	
IV	2	421.784	
T classification			
T1	5	9.978	0.000
T2–T3	21	29.235	
T4	4	399.789	
N classification			
N0	16	10.44	0.001
N1	14	21.29	
Differentiation			
well	13	17.335	0.006
moderate	11	24.433	
poor	6	48.712	

### High Expression Levels of HOTAIR Correlate with Poor Prognosis in ESCC Patients

**Figure 3:**
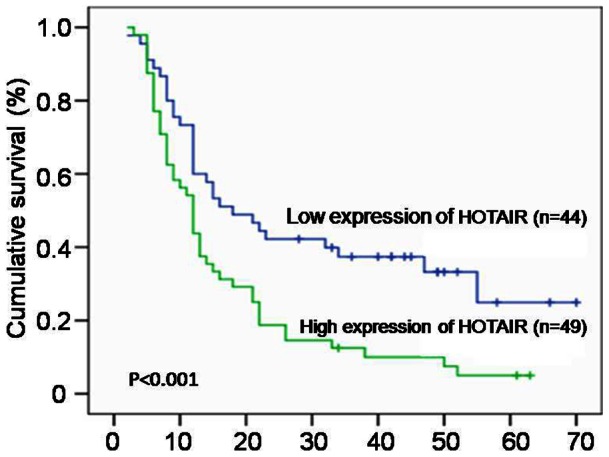
Overall survival curves for ESCC patients with high or low expression of HOTAIR. Patents expressing high level of HOTAIR have a significantly shorter survival.

The median survival of patients with low and high HOTAIR expression levels was 18 months and 12 months, respectively. The 5-year survival rates of ESCC patients with high HOTAIR expression levels (5.0%) were significantly lower than those of patients with low HOTAIR expression levels (24.9%; *P*<0.001, Figure 3). A multivariate Cox regression analysis showed that the level of HOTAIR expression was an independent factor for overall survival (RR = 1.985, *P* = 0.005).

### HOTAIR Regulates the Proliferation and Colony Formation of ESCC Cells

**Figure 4:**
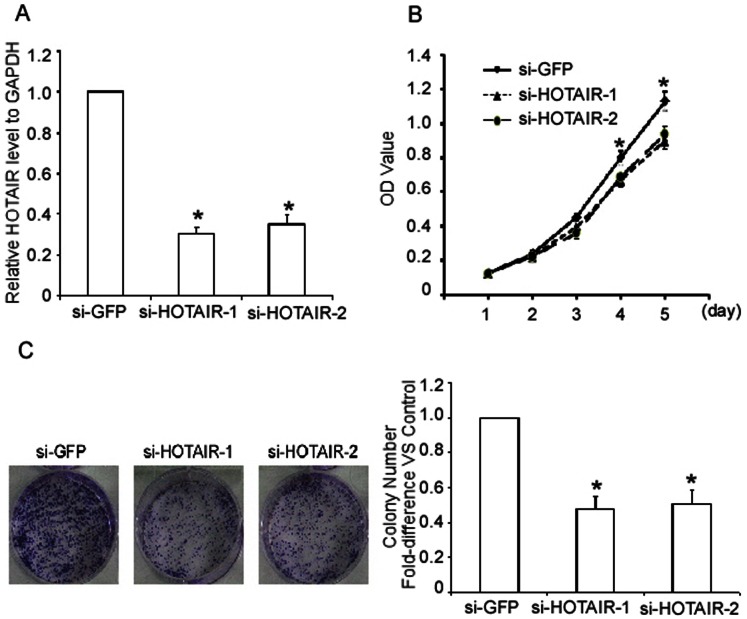
Silencing of HOTAIR suppresses TE-1 cell growth and colony formation. A. Transfection with all siRNAs potently reduced the target mRNA levels. TE-1 cells were transfected with siRNAs against HOTAIR and GFP for 48 h. HOTAIR mRNA levels were evaluated by real-time quantitative PCR. GAPDH was used as an internal control. B. Silencing of HOTAIR suppressed the proliferation of TE-1 cells as determined by an MTT assay. TE-1 cells that were transfected with siRNAs against HOTAIR or GFP for the indicated times were subjected to an MTT assay. C. The effect of HOTAIR on the tumorigenesis of TE-1 cells was examined by a colony formation assay. The results are expressed as the means ± SD; n = 3, * *P*<0.01 compared with the negative control.

Our clinical data indicated that HOTAIR expression levels correlated inversely with ESCC progression. Therefore, we examined whether HOTAIR regulated the proliferation of ESCC cells. siRNAs targeting HOTAIR substantially down-regulated HOTAIR level (Figure 4A). Silence of HOTAIR notably repressed the growth of TE-1 cells compared with the negative control (Figure 4B), indicating that HOTAIR may promote ESCC cell proliferation. Furthermore, in a colony-forming assay, the numbers of HOTAIR-silenced TE-1 cell colonies were reduced approximately 2-fold compared with the negative control (Figure 4C).

### HOTAIR Regulates the Migration of ESCC Cells

**Figure 5:**
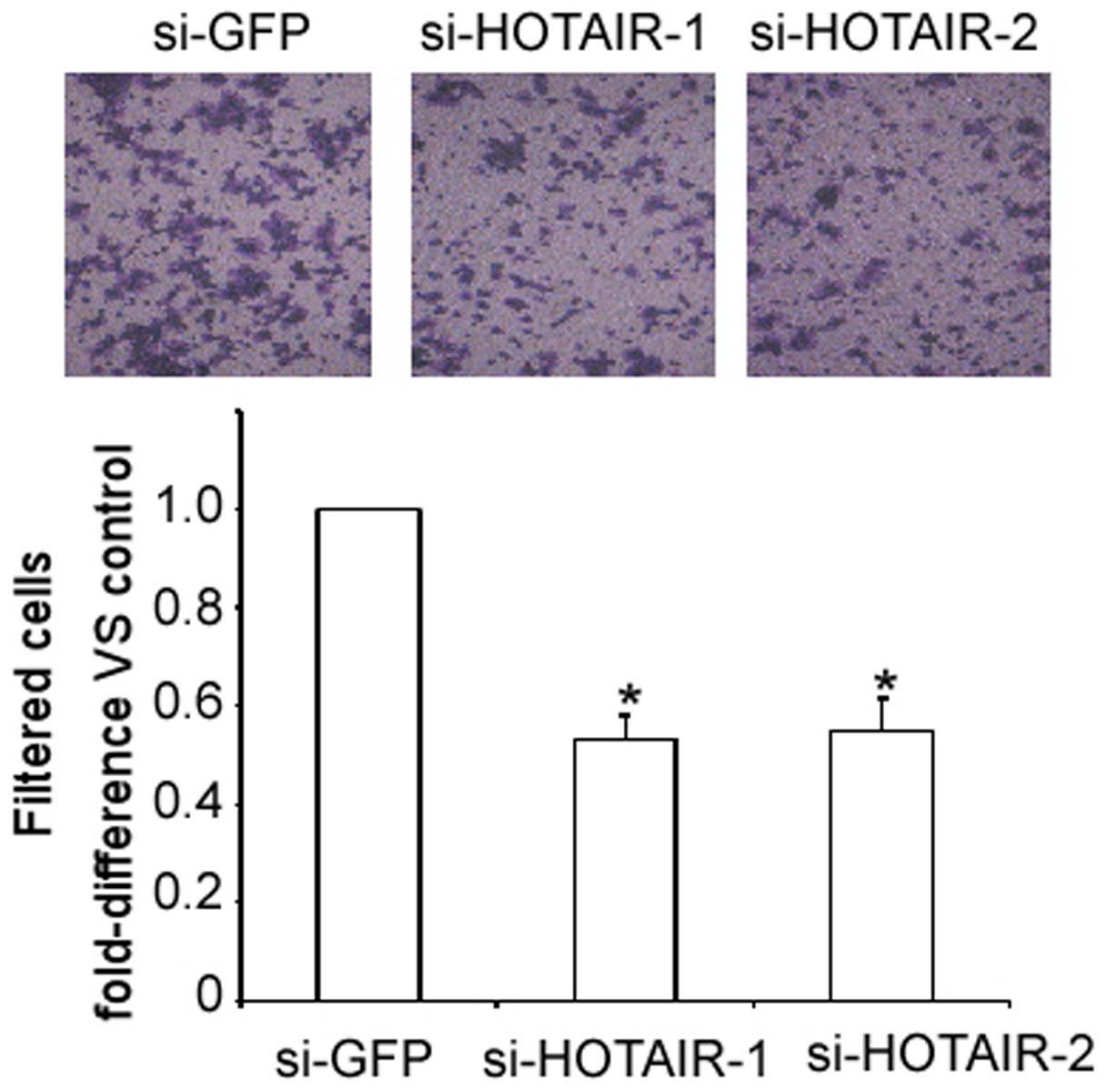
Silencing of HOTAIR suppresses the migratory capacity of TE-1 cells as determined by a transwell assay. TE-1 cells that were transfected with siRNAs against HOTAIR or GFP for 48 h were subjected to a transwell assay. The numbers of filtered cells were counted by microscopy. The results are expressed as the means ± SD; n = 3, * *P*<0.01 compared with the negative control.

We also assessed whether HOTAIR mediated the migration of ESCC in vitro through the use of Boyden chamber assays. HOTAIR silencing reduced the number of migrating TE-1 cells by approximately 50% compared with the negative control (Figure 5). These data suggest that HOTAIR plays a critical role in mediating ESCC progression.

## Discussion

In this study, we found that high expression levels of HOTAIR, a cancer-related lncRNA, correlated clinically with ESCC progression. HOTAIR is a predictor of ESCC patient prognosis. Additionally, HOTAIR contributes to the malignant phenotype of ESCC cells through its regulation of diverse cellular processes, including migration, invasion and proliferation.

Recently, roles for lncRNAs as drivers of tumor suppressive and oncogenic functions have been suggested in various cancer types. HOTAIR has been reported to associate with PRC2 and to epigenetically regulate multiple target genes [12]. HOTAIR has been demonstrated to be upregulated in breast cancer, hepatocellular carcinoma, pancreatic cancer, laryngeal squamous cell carcinoma and colorectal cancer [12], [15], [16], [18], [20]–[23], [25], [26]. In this study, we identified increased HOTAIR levels in ESCC tissues versus non-cancerous tissues by ISH and qRT-PCR. Additionally, the expression levels of HOTAIR were upregulated in samples from patients with higher tumor burdens, who were defined as those with larger tumors, advanced clinical staging, increased lymph node tumor burdens and the presence of distant metastases. These data indicate that increased HOTAIR expression levels are associated with the progression of ESCC. Moreover, we studied the relationship between HOTAIR expression and patient prognosis and discovered that high expression levels of HOTAIR in ESCC corresponded remarkably to patient survival. Our results suggest that high expression levels of HOTAIR may play an important role in the development, tumorigenesis and progression of ESCC.

Emerging evidence suggests that HOTAIR regulates the proliferation and metastasis of a variety of tumor cells. The suppression of HOTAIR in liver cancer cells reduces cell viability and invasion, sensitizes the cells to TNF-induced apoptosis and increases chemotherapeutic sensitivity [26]. In breast cancer, the overexpression of HOTAIR increases metastatic potential both in vitro and in vivo [12]. The knockdown of HOTAIR suppresses the invasiveness of gastrointestinal stromal tumor cells [22]. Similarly, our data demonstrated that the silencing of HOTAIR suppressed the proliferation, migration and invasion of ESCC TE-1 cells. Thus, these in vitro experiments suggest that HOTAIR may regulate the progression of ESCC cells, although the mechanisms remain to be explored.

In conclusion, the results of our study indicate that the expression of HOTAIR correlates strongly with the clinical stages and overall survival times of ESCC patients and that the upregulation of HOTAIR plays an important role in ESCC progression. However, further studies are needed to investigate the underlying mechanisms of HOTAIR in the regulation of ESCC progression. Such studies have the potential to provide novel therapeutic strategies for ESCC treatment.
